# Evaluation of Highly Detectable Pesticides Sprayed in *Brassica napus* L.: Degradation Behavior and Risk Assessment for Honeybees

**DOI:** 10.3390/molecules23102482

**Published:** 2018-09-27

**Authors:** Zhou Tong, Jinsheng Duan, Yancan Wu, Qiongqiong Liu, Qibao He, Yanhong Shi, Linsheng Yu, Haiqun Cao

**Affiliations:** 1 Institute of Plant Protection and Agro-Product Safety, Anhui Academy of Agricultural Sciences, Hefei 230031, China; tongzhou0520@163.com (Z.T.); djszbzas@126.com (J.D.); 2 Key Laboratory of Agro-Product Safety Risk Evaluation (Hefei), Ministry of Agriculture, Hefei 230031, China; 3 School of Plant Protection, Anhui Agricultural University, Hefei 230036, China; wuyancan1989@163.com (Y.W.); ahbb1104@163.com (Q.L.); heqibao0418@126.com (Q.H.); 4 Hefei Testing and Inspection Center for Agricultural Products Quality, Hefei 230601, China; 5 School of Resource & Environment, Anhui Agricultural University, Hefei, 230036, China; shiyh@ahau.edu.cn; 6 School of Animal Science and Technology, Anhui Agricultural University, Hefei 230036, China; yulinsheng@ahau.edu.cn

**Keywords:** pesticide, honeybee, risk, field

## Abstract

Honeybees are major pollinators of agricultural crops and many other plants in natural ecosystems alike. In recent years, managed honeybee colonies have decreased rapidly. The application of pesticides is hypothesized to be an important route leading to colony loss. Herein, a quick, easy, cheap, effective, rugged, and safe (QuEChERS) method was used to determine eight highly detectable pesticides (carbendazim, prochloraz, pyrimethanil, fenpropathrin, chlorpyrifos, imidacloprid, thiamethoxam, and acetamiprid) in rape flowers. A field experiment was conducted at the recommended dose to evaluate the contact exposure risk posed to honeybees for 0–14 days after treatment. The initial residue deposits of neonicotinoids and fungicides among these compounds were 0.4–1.3 mg/kg and 11.7–32.3 mg/kg, respectively, and 6.4 mg/kg for fenpropathrin and 4.2 mg/kg for chlorpyrifos. The risk was quantified using the flower hazard quotient (FHQ) value. According to the data, we considered imidacloprid, thiamethoxam, chlorpyrifos, fenpropathrin, and prochloraz to pose an unacceptable risk to honeybees after spraying in fields, while fungicides (carbendazim and pyrimethanil) and acetamiprid posed moderate or acceptable risks to honeybees. Therefore, acetamiprid can be used instead of imidacloprid and thiamethoxam to protect rape from some insects in agriculture, and the application of prochloraz should be reduced.

## 1. Introduction

The honeybee is a social insect species. It plays a major environmental role by providing necessary ecosystem services, similar to bumblebees, solitary bees, moths, butterflies, beetles, and other insects [1]. As the most abundant pollinators globally, honeybees contribute an estimated €153 billion annually to global agriculture [2]. However, in recent decades, managed honeybee colonies have decreased in North America, Europe, and Asia-Pacific [3,4,5,6,7,8,9], known as colony collapse disorder (CCD). Presently, honeybees are exposed to diverse stressors, including Varroa mites (*Varroa destructor*) and viruses [10], pathogens [11], pesticides [12,13], habitats [14] and climate change [15].

Sublethal doses of pesticides have been proven to disrupt memory, learning, navigation, and foraging activities in honeybees, and affect their sensitivity to other stressors, particularly increasing pathogen vulnerability [16,17,18,19]. Concern about the risk posed to bees by pesticides not only includes the toxicity evaluation of plant protection products, but also accurate estimation of the residue exposure levels from these products in the environment. With the development of analysis technique, the quick, easy, cheap, effective, rugged, and safe (QuEChERS) approach and UPLC–MS/MS have extensive application. Some complex matrices, such as soil [20], meat product [21] et al. were extracted through the QuEChERS. The high sensitivity and selectivity have also been provided by ultra-high-performance liquid chromatography–tandem mass spectrometry (UPLC–MS/MS) [22] and gas chromatography–tandem mass spectrometry (GC–MS/MS) [23]. Meanwhile, a large number of bee product was monitored in decade. For example, pesticides have been detected in pollen, beeswax, and honeybee samples collected in Italy [24,25,26]. A survey conducted on apiaries in France found that honeybees are exposed to multiple miscellaneous pesticides simultaneously from other honeybees, pollen, bee bread, and beehives [27,28,29,30,31,32]. In Poland, 48 different pesticide residues have been found in honeybees [33,34,35]. Also, other studies on the influence of 5 different pesticides on honeybees were conducted in Poland [36]. China is a vast region with a complex climate that has a long history of honeybee management. In rapeseed crops in China, eight pesticides, namely carbendazim, prochloraz, pyrimethanil, fenpropathrin, chlorpyrifos, imidacloprid, thiamethoxam, and acetamiprid, have been detected at high residue levels in pollen samples by UPLC–MS/MS [37]. Therefore, risk assessment of these chemicals is increasingly important to protect pollinators from the adverse effects of plant protection products (PPPs).

Risk assessments for honeybees typically consider only the acute toxicity of pesticides either through topical or oral exposure for 24 or 48 h, ignoring the negative effects resulting from sustained exposure to pesticide residues over longer periods. Some evaluations have paid attention to the application rates of different chemicals to estimate a hazard quotient (HQ) [38]. In the case of environmental risk assessments for honeybees, exposure assessments have been defined in general terms. Toxicity data is combined with contact and dietary exposure, and calculated separately using two approaches that are specific to different applications. Since 2010, individual studies have used pesticide residues in a plant matrix (pollen, nectar, or the aerial part of the plant) to evaluate the toxicity exposure ratio (TER) [39] or pollen hazard quotient (PHQ) [40]. As proposed in the European and Mediterranean Plant Protection Organization (EPPO)’s document, ‘Environmental risk assessment scheme for plant protection products Chapter 10: honeybees’, such residues to honeybees occur through both contact with and ingestion of plant protection products. Two tiers of risk assessment are included in this document, with the primary procedure evaluating the HQ or TER in the laboratory, while field trials are proposed for a high tier risk assessment.

This study aims to assess the risk posed by some highly detectable pesticides, such as carbendazim, prochloraz, pyrimethanil, fenpropathrin, chlorpyrifos, imidacloprid, thiamethoxam, and acetamiprid [37] to honeybees following spraying in rape fields. After treatment on specific days, it is necessary to evaluate the exposure risk of these compounds, which are widely used to protect crops against aphids, *Plutella xylostella* (L.), *Sclerotinia sclerotiorum (Lib.) de Bary*, and *Peronospora parasitica (Pers.) Fr.*, to legitimately guide pesticide application and honeybee management. Pesticide residues on rape flowers were used in the exposure to calculate the flower hazard quotient (FHQ) in order to estimate the risk of contact with pollinators after pesticide spraying.

## 2. Materials and Methods

### 2.1. Chemicals and Standards

A Milli-Q ultrapure water system manufactured by Millipore (Milford, UT, USA) was used throughout experiments to provide the HPLC-grade water that was employed during analysis and to hydrate the rape flower samples. Ultragradient HPLC-grade acetonitrile and methanol were obtained from Tedia (Shanghai, China). Formic acid, acetic acid, anhydrous magnesium sulfate (MgSO_4_), and sodium acetate (NaOAc) were supplied by Sinopharm Chemical Reagent Co., Ltd. (Shanghai, China). PSA (primary and secondary amine-bonded silica), C_18_ (octadecyl-bonded silica) and GCB (graphitized carbon black) were purchased from Agilent Technologies (Santa Clara, CA, USA). 

Reference standards of imidacloprid (purity, 98%), thiamethoxam (purity, 95%), acetamiprid (purity, 95%), fenpropathrin (purity, 98.5%), chlorpyrifos (purity, 98%), carbendazim (purity, 95%), prochloraz (purity, 98%) and pyrimethanil (purity, 95%) were obtained from Dr. Ehrenstorfer GmbH (Augsburg, Germany).

Formulated pesticides of imidacloprid 10% wettable powder (WP), thiamethoxam 25% water-dispersible granules (WDG), acetamiprid 20% WP, fenpropathrin 20% emulsifiable concentrate (EC), chlorpyrifos 40% EC, carbendazim 80% WP, prochloraz 25% EC, and pyrimethanil 40% suspension concentrate (SC) were purchased from the agricultural market located in Hefei, Anhui, China.

### 2.2. Experimental Design and Sample Collection

Field experiments of each chemical were conducted using the same rape variety at the Experimental Station of Anhui Agriculture University, Anhui, China, in accordance with good agricultural practices (GAP). At each site, 3 m × 30 m rectangular plots spaced 1 m apart were flagged at the corners. Three replicate plots were designated for each pesticide, in addition to an untreated control plot. No mixtures were used on individual experimental plots. Spray application of the chemicals was achieved using an automatic sprayer at the recommended dose (shown in Table 1, Table 2 and Table 3) during rape flowering. The formulated compounds were dissolved in water, with 900 L/ha of water used for application. Rape flower samples were collected from five stochastically assigned subsections in each plot and combined into a plastic bag for each replicate. Flower sampling from each control was repeated 0, 1, 3, 5, 7, 10, and 14 days after spraying. The information of meteorology was listed in Table 4. All the samples were frozen immediately and stored at −20 °C until extraction for residue determination (up to 15 days). The storage stability tests were conducted before the sample analysis, and the results proved that the degradation rate of all the target compounds storing in −20 °C was lower than 5% entirely.

### 2.3. Sample Preparation

Rape flower samples were grated with liquid nitrogen and 2 g of the sample were weighed into a 50 mL polypropylene centrifuge tube. Ultrapure water (3 mL) was then added and the tube was shaken for 30 s to hydrate the sample. Next, 0.1 N acetic acid in acetonitrile (10 mL) and glass beads (2 g) were added and the samples were vortexed for 2 min at room temperature, followed by freezing for 10 min at −20 °C in a freezer. MgSO_4_ (0.5 g) and NaOAc (2 g) were then added and the tube was shaken vigorously for 60 s and centrifuged for 5 min at 3800 rpm. A 5 mL aliquot of the supernatant (acetonitrile phase) was transferred to a 15-mL centrifuge tube containing the QuEChERS (Quick, Easy, Cheap, Effective, Rugged, and Safe) salt kit (1.25 g; PSA:C18:MgSO_4_:GCB, 1:1:3:0.15 (*w*/*w*/*w*/*w*)). After mixing well, the mixture was again centrifuged at 3800 rpm for 5 min, and 2.5 mL of the acetonitrile phase was transferred to a glass tube. The supernatant was then evaporated to dryness under a gentle stream of nitrogen at 30 °C. The dry residue was dissolved in methanol (0.5 mL, HPLC grade), filtered through a 0.22 μm filter membrane, and introduced into an autosampler vial for UPLC–MS/MS analysis.

### 2.4. Chemical Analysis

A Waters Acquity ultra-performance liquid chromatography system (Waters, Milford, MA, USA) equipped with a binary pump and Acquity EBH (ethylene bridged hybrid) C_18_ column (1.7 μm i.d., 2.1 mm × 100 mm particle size) was used for liquid chromatography. Components were separated using Milli-Q water/methanol (98:2) + 0.05 N formic acid (solvent A) and methanol + 0.05 N formic acid (solvent B) at 40 °C. Separation was performed with a flow rate of 0.45 mL/min starting with a mobile phase of 5% B in A at the injection time, which was then rapidly increased to 100% B over 0.25 min and then held at 100% mobile phase B until 8.5 min. Finally, the mobile phase was switched to 5% B in A at 8.51 min and held until 10 min to re-equilibrate the column. The injection volume was 3 μL and the total analytical time was 10 min.

A Waters Xevo TQ triple quadrupole mass spectrometer (Waters, Milford, MA, USA) equipped with an electrospray ionization (ESI) source and conducted in positive electrospray ionization mode was used for mass spectrometry detection. The ESI source temperature was 150 °C. The gas flow rates of the cone and desolvation gases were 50 and 900 L/h. The desolvation temperature was 500 °C. Helium was used as the collision gas with a flow rate of 0.15 mL/min. The mass spectrometer was operated in multiple reaction monitoring (MRM) mode to monitor two precursor ion transitions for each pesticide. The target ion transition with the highest response (primary ion transition) was used for quantification and the second target ion transition was used for confirmation [41]. Confirmation was provided through a product ion scan (PIC) of each peak, which adapted to a reference spectrum for each analysis. The quantification and confirmation calculations were calculated using Target Lynx 4.1 software (Waters Corp., Milford, MA, USA) implemented by the apparatus. Other MS settings used in this experiment, including ion transitions, cone voltages, collision energies, and dwell times, are shown in Table S1.

### 2.5. Preparation of Standard Solutions

All standard stock solutions (1000 ng/mL) were prepared in acetonitrile, and further diluted to prepare working standards. Calibration standards (1–100 ng/mL) were prepared by diluting the working standards. Standards with concentrations in the range 1–100 ng/mL were prepared for spiking blank rape flowers. Matrix-matched standards were prepared by adding the pesticides to the blank sample. Blank sample (1 mL) extract was evaporated to dryness under nitrogen, and the required calibration standard (1 mL) was added to prepare matrix-matched standards of the required concentrations (1–100 ng/mL).

### 2.6. Data Analysis

The limit of detection (LOD) was determined as the minimum concentration detectable for all target chemicals based on a signal-to-noise (S/N) ratio of 3, while the limit of quantification (LOQ) was set as a S/N ratio of 10 [42].

Matrix effects (ME) were calculated from the slopes of calibration curves in the matrix and solvent for each determination using Equation (1).

(1) $$ME\left(\%\right)=\left(\left(\frac{Slope\;of\;calibration\;on\;curve\;in\;matrix}{Slope\;of\;calibration\;on\;curve\;in\;solvent}\right)-1\right)\times 100$$

To evaluate the risk posed to honeybees by highly detectable pesticides, we used distinct new approaches to assess oral exposure. For exposure through the uptake of pollen or honey from the flower, the oral LD50s shown in Table S2 were used to calculate the daily oral flower hazard quotient (FHQ_do_) for honeybees exposed to 1 g of contaminated flowers per day [43].

(2) FHQdo=PEC(ng/g)×MCL(g)LoralD50(ng/bee)

Using Equation (2), the FHQ_do_ was calculated from the oral exposure dose (equal to the predicted exposure concentration (PEC) multiplied by the maximum contact level (MCL)) and the acute oral LD50 for adult bees. When the FHQ_do_ value was lower than 0.1, the risk was considered acceptable, while values between 0.1 and 1 indicated moderate risks, and values greater than 1 indicated an unacceptable risk.

## 3. Results and Discussion

### 3.1. Method Validation

The LOD and LOQ were calculated from the regression data shown in Table S3. The LODs ranged from 0.0088 to 0.1064 ng/mL. Calibration curves, including the zero point, were constructed using the blank rape flower matrix spiked at six different concentration levels in the range 1–100 ng/mL after the extraction step. This calibration could reduce the impact of matrix effects in the electrospray source, such as ion enhancement or suppression. The linear region, observed throughout the concentration range studied depending on the chemicals, is shown in Table S3. Good linearity was observed in all cases, with correlation coefficients (R^2^) better than 0.9902, which assumed the quantitative analysis of pesticide residues in rape flower.

During MS analysis, quantification was subject to strong matrix effects (ME) that could severely reduce or promote the response of the chemical. Therefore, the ME of each compound is described in Table 1. Among the eight pesticides, unacceptable matrix effects were observed in the range of −34% to 75%. To reduce matrix effects, we prepared analytical curves in the matrix.

Recovery studies of the eight pesticides were performed in rape flower samples at three spiked levels of 5, 50, and 500 ng/g. The accuracy and precision of the analytical method were evaluated using these studies. The recoveries and relative standard deviation (RSD) are listed in Table 5. Recoveries obtained for all chemicals ranged from 78.9% to 115.2%, with all pesticides within the satisfactory range of 70–120%. The RSD value was included in RSD_r_ and RSD_R_, which calculated using the standard deviation of the recovery on the same day and the three separate days, respectively. In this experiment, the RSD_r_ values ranged from 1.2% to 10.6%. Meanwhile, the RSD_R_ values ranged from 3.0% to 11.2%.

### 3.2. Residues of Highly Detectable Pesticides on Rape Flowers

**Figure 1:**
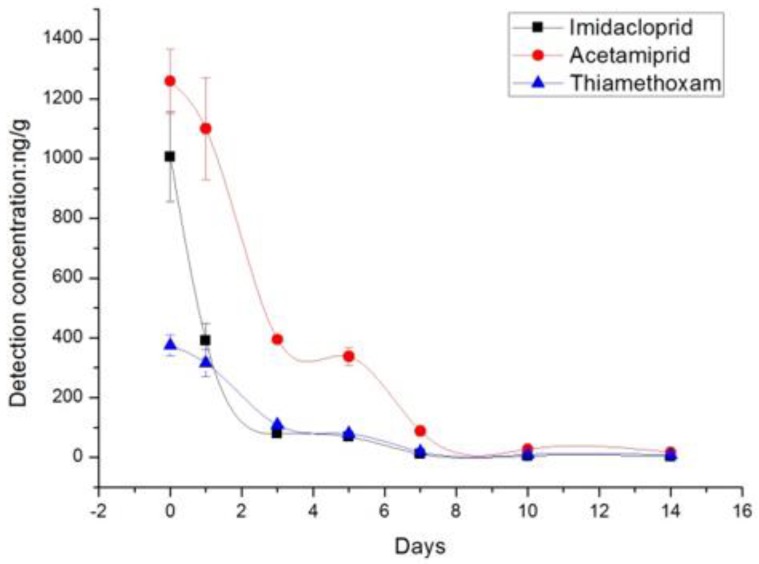
Residue dissipation of the Neonicotinoids on rape flower.

As shown in Table 1, the average initial residue deposits of the neonicotinoids were 370.0, 1006.0, and 1259.0 ng/g from treatments with thiamethoxam, imidacloprid, and acetamiprid at the recommended doses, respectively. On day 1 after treatment, 12.7–15.9% of the neonicotinoids residues had dissipated from the rape flower, which increased to 93.1–95.0% by day 7, with the exception of imidacloprid, which showed substantial degradation (61.2%) on day 1. Imidacloprid residues dissipated fast in the initial stages. On day 5 after application, more than 70% of the residues had dissipated from both treatments, which increased to about 95% by day 10. On day 10, the residue levels of imidacloprid were below the LOQ. The dissipation trend of the neonicotinoids is described in Figure 1.

**Figure 2:**
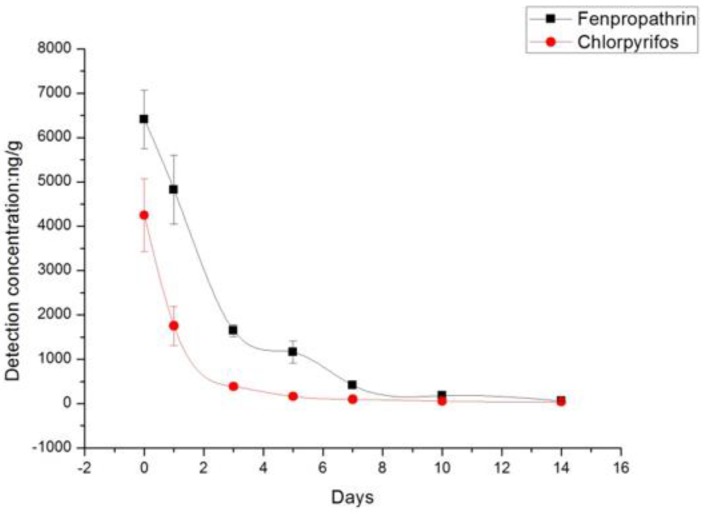
Residue dissipation of chlorpyrifos and fenpropathrin on rape flower.

As shown in Table 2, the average initial residue deposits of chlorpyrifos and fenpropathrin were 4249 ng/g and 6409 ng/g, respectively, after treatment at the recommended doses. On day 1 after treatment, 24.7% of fenpropathrin residues had dissipated from the rape flower, which increased to 93.6% by day 7. In contrast, 58.7% of chlorpyrifos residues had dissipated from the rape flower by day 1, which increased to 90.9% by day 3. On day 14, the residue levels of chlorpyrifos and fenpropathrin were 38.7 ng/g and 58.7 ng/g. The dissipation trends of chlorpyrifos and fenpropathrin are described in Figure 2, showing that the degradation rate of chlorpyrifos was much faster than that of fenpropathrin.

**Figure 3:**
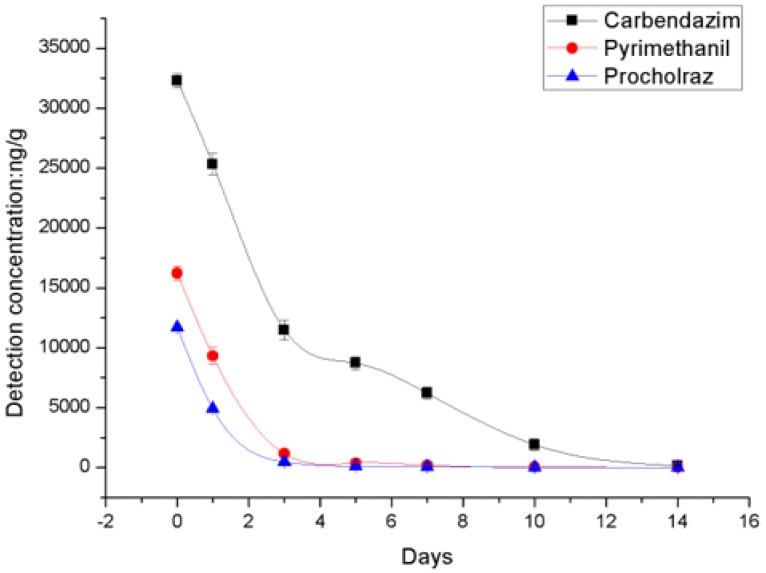
Residue dissipation of the Fungicides on rape flower.

As shown in Table 3, the average initial residue deposits of the fungicides were 32,299 ng/g, 16,222 ng/g, and 11,684 ng/g from treatments with carbendazim, pyrimethanil, and prochloraz, respectively, at the recommended doses. On day 1, 42.6–58.1% of the fungicide residues had dissipated from the rape flowers, which increased to 92.9–96.0% by day 3, with the exception of carbendazim, which showed relatively little degradation (21.8%) after day 1. Pyrimethanil and prochloraz residues dissipated rapidly during the initial stages. On day 5 after application, more than 70% of carbendazim residues had dissipated, which increased to about 90% by day 10. On day 10, the prochloraz residue level was below the LOQ. The dissipation trend of the fungicides is described in Figure 3, showing that the degradation rate of carbendazim was much slower than those of pyrimethanil and prochloraz.

### 3.3. Risk Assessment

To understand the risks posed to honeybees by the application of highly detectable pesticides, FHQ_do_ values (shown in Table 6) were calculated using Equation (1), as shown in the ‘Data analysis’ section. Risks were classed as acceptable, moderate, and unacceptable. 

Among the neonicotinoids, the risk was unacceptable for all, except acetamiprid, up to day 14 after treatment. Meanwhile, imidacloprid retained the most harmful oral exposure risk. The oral risk posed by acetamiprid was much lower than those of imidacloprid and thiamethoxam, and classed as acceptable. In Jiang’s research, imidacloprid and thiamethoxam have been detected and respectively ranged from 1.61 to 64.58 ng/g and ND (not detected) to 31.52 ng/g in cotton pollen after seed treatment [22]. It almost seemed that the neonicotinoids residue level of spray treatment is higher than seed treatment. Meanwhile, the risk to honeybees was unacceptable equally. Chlorpyrifos also carried an unacceptable risk level until day 3 after spraying, after which the risk decreased to moderate until day 14. Through data analysis, we considered the insecticides, comprising imidacloprid, thiamethoxam, and chlorpyrifos, to have a high oral exposure risk when used for aphid treatment, with the exception of acetamiprid. Therefore, when using plant protection products to prevent aphids on flowering rape, honeybee management could be achieved using acetamiprid instead of imidacloprid, thiamethoxam, and chlorpyrifos.

Fenpropathrin is a major chemical used to protect flowering rape against insects such as *Plutella xylostella* (L.) [44]. On the spraying day, the FHQ value reached 128.18, and remained at an unacceptable exposure risk level for honeybees until day 14 after treatment. Therefore, honeybee management will be improved at least 14 days after spraying with fenpropathrin.

Plant diseases, such as *Sclerotinia sclerotiorum (Lib.) de Bary* and *Peronospora parasitica (Pers.) Fr*, are a primary cause of oilseed rape destruction during growth. To prevent such diseases, a large number of fungicides, including carbendazim, pyrimethanil, and prochloraz, are widely used in agriculture. As shown in Table 5, the fungicides posed lower risks, with the exception of prochloraz, for which the unacceptable risk to honeybees was sustained until day 5 after treatment, and became acceptable after day 10. As the recommended dose of carbendazim was large, a moderate risk to honeybees remained until day 7 after treatment. For pyrimethanil, a moderate risk to honeybees was present on day 1 after treatment. Although the fungicides, including carbendazim and pyrimethanil, were found to pose only moderate risks to honeybees when sprayed in rape fields, recent studies have shown that fungicides have a negative effect on honeybee colonies [45]. This effect is probably due to the synergistic toxic effects that certain fungicides, specifically azoles and prochloraz, have on insects such as honeybees [46].

## 4. Conclusions

The degradation rates of pesticide residues of eight highly detectable chemicals sprayed in an oilseed rape field were generally rapid. However, some high toxicity insecticides, including imidacloprid, thiamethoxam, chlorpyrifos, and fenpropathrin, posed unacceptable oral exposure risks to honeybees. The three fungicides assessed posed moderate risks to honeybees, with the exception of prochloraz, which produced an unacceptable risk more than five days after treatment. This showed that the logical application of pesticides is integral to honeybee management. In the future work, we have designed to determinate pesticide residues in other crop species, and risk assessment on honeybee larva would be investigated combining more toxicology data.

## Figures and Tables

**Table 1:** Residues of neonicotinoids in rape flowers.

Days after Treatment	Untreated Control	Residue Levels of the Neonicotinoids (ng/g) ± SD					
		Thiamethoxam (30 g a.i./ha) ME (29%)		Imidacloprid (60 g a.i./ha) ME (75%)		Acetamiprid (60 g a.i./ha) ME (−5%)	
		Residue	Degradation Rate (%)	Residue	Degradation Rate (%)	Residue	Degradation Rate (%)
0 day	ND	375.0 ± 35.5	-	1006.0 ± 150.3	-	1259.0 ± 108.2	-
1 day	ND	315.3 ± 45.3	15.9	390.0 ± 57.5	61.2	1099.7 ± 170.4	12.7
3 day	ND	108.7 ± 3.1	71.0	78.7 ± 1.5	92.2	394.3 ± 8.6	68.7
5 day	ND	78.7 ± 7.2	79.0	68.3 ± 3.1	93.2	337.7 ± 29.3	73.2
7 day	ND	18.7 ± 2.5	95.0	11.3 ± 1.2	98.9	87.3 ± 5.5	93.1
10 day	ND	9.3 ± 1.2	97.5	ND	-	27.3 ± 1.5	97.8
14 day	ND	8.0 ± 1.0	97.9	ND	-	10.3 ± 0.6	98.7
DT50 day	-	2.3		1.2		1.9	

**Table 2:** Residues of chlorpyrifos and fenpropathrin in rape flowers.

Days after Treatment	Untreated Control	Residue Levels of Insecticides (ng/g) ± SD			
		Chlorpyrifos (360 g a.i./ha) ME (22%)		Fenpropathrin (60 g a.i./ha) ME (−8%)	
		Residue	Degradation Rate (%)	Residue	Degradation Rate (%)
0 day	ND	4249 ± 821.1	-	6409 ± 656.6	-
1 day	ND	1755 ± 441.6	58.7	4824.± 778.7	24.7
3 day	ND	386.3 ± 14.3	90.9	1646 ± 129.5	74.3
5 day	ND	160.7 ± 15.6	96.2	1159 ± 246.0	81.9
7 day	ND	98.3 ± 14.0	97.7	412.0 ± 66.8	93.6
10 day	ND	57.3 ± 4.9	98.7	179.0 ± 21.1	97.2
14 day	ND	38.7 ± 5.5	99.1	58.7 ± 11.5	99.1
DT50 day	-	2.1		2.0	

**Table 3:** Residues of the fungicides in rape flowers.

Days after Treatment	Untreated Control	Residue Levels of the Fungicides (ng/g) ± SD					
		Carbendazim (1500 g a.i./ha) ME (50%)		Pyrimethanil (360 g a.i./ha) ME (−34%)		Prochloraz (181.5 g a.i./ha) ME (−20%)	
		Residue	Degradation Rate (%)	Residue	Degradation Rate (%)	Residue	Degradation Rate (%)
0 day	ND	32,299 ± 583.0	-	16,222 ± 573.7	-	11,684 ± 167.0	-
1 day	ND	25,316 ± 878.0	21.6	93,089 ± 724.9	42.6	4891 ± 150.3	58.1
3 day	ND	11,478 ± 814.5	64.5	1155 ± 104.4	92.9	471.7 ± 58.1	96.0
5 day	ND	8713 ± 564.2	73.0	374.3 ± 23.6	97.7	100.3 ± 4.7	99.1
7 day	ND	6196 ± 498.3	80.8	195.7 ± 2.1	98.8	57.3 ± 0.6	99.5
10 day	ND	1908 ± 477.6	94.1	50.3 ± 3.2	99.7	11.3 ± 2.9	99.9
14 day	ND	153.3 ± 17.9	99.5	9.3 ± 1.5	99.9	ND	-
DT50 day		2.0		1.3		1.0	

**Table 4:** The information of meteorology during the trial days.

Days after Treatment	0 Day	1 Day	3 Day	5 Day	7 Day	10 Day	14 Day
Data	27 March	28 March	30 March	1 April	3 April	6 April	10 April
Season	Spring						
Weather	Sunny	Sunny	Cloudy	Rain	Cloudy	Cloudy	Sunny
Rainfall	-	-	-	4 mm	-	-	-
Temperature	24 °C	25 °C	22 °C	22 °C	21 °C	25 °C	26 °C

**Table 5:** Recoveries, relative standard deviations (RSDs), and matrix effects (MEs) of eight pesticide compounds in rape flowers.

Compound	ME	Spiked Level (ng/g)	Intra-Day (*n* = 5)						(Inter-Day) (*n* = 15) RSD_R_ (%)
			Day 1		Day 2		Day 3		
			Recovery (%)	RSD_r_ (%)	Recovery (%)	RSD_r_ (%)	Recovery (%)	RSD_r_ (%)	
Carbendazim	50	5	95.6	4.7	88.9	3.9	90.5	6.9	5.2
		50	88.4	3.6	78.9	2.2	92.6	6.5	4.1
		500	97.6	3.2	88.5	5.7	91.8	6.2	5.0
Thiamethoxam	29	5	100.5	1.2	97.6	5.0	95.3	4.6	3.6
		50	94.3	4.8	95.3	4.2	92.8	4.0	3.0
		500	97.1	2.4	93.2	2.8	115.2	6.1	3.8
Imidacloprid	75	5	95.7	4.2	88.4	9.7	90.6	6.3	4.7
		50	88.4	1.2	93.4	2.3	93.7	5.6	3.0
		500	96.5	2.9	93.2	5.2	95.6	4.9	4.3
Acetamiprid	−5	5	100.1	4.6	95.6	4.9	93.2	6.1	5.2
		50	91.2	2.1	105.2	5.6	92.9	4.8	4.2
		500	98.0	5.6	94.2	4.2	89.9	5.9	5.2
Pyrimethanil	−34	5	88.9	3.6	94.2	3.8	89.5	8.9	5.4
		50	89.7	4.2	91.3	4.9	90.8	5.4	4.8
		500	90.8	5.2	96.5	3.1	92.7	5.6	3.0
Procholraz	−20	5	95.7	4.2	88.4	3.7	90.6	6.3	4.7
		50	88.4	1.2	93.4	2.3	93.7	5.6	3.0
		500	96.5	2.9	93.2	5.2	95.6	4.9	4.3
Chlorpyrifos	22	5	100.1	4.6	95.6	4.9	93.2	6.1	11.2
		50	91.2	2.1	95.2	5.6	92.9	4.8	4.2
		500	98.0	10.6	94.2	4.2	89.9	5.9	5.2
Fenpropathrin	−8	5	92.3	4.8	99.2	4.5	91.2	6.2	5.2
		50	89.6	2.5	92.5	3.7	91.8	6.5	4.2
		500	95.2	6.6	94.6	2.5	88.3	5.4	4.8

**Table 6:** Risk level of eight pesticides following spraying in a rape field.

Days after Treatment	Oral Flower Hazard Quotient (FHQ_do_)							
	Thiamethoxam	Imidacloprid	Acetamiprid	Chlorpyrifos	Fenpropathrin	Carbendazim	Pyrimethanil	Prochloraz
0 day	75.02	773.78	0.09	17.71	128.18	0.65	0.32	116.80
1 day	63.15	300.21	0.08	7.31	96.48	0.51	0.19	48.91
3 day	21.76	60.76	-	1.61	32.91	0.23	0.02	4.72
5 day	15.42	52.49	-	0.67	23.19	0.17	-	1.03
7 day	3.72	8.74	-	0.41	8.24	0.12	-	0.57
10 day	1.91	3.61	-	0.24	3.58	0.04	-	0.11
14 day	1.62	2.38	-	0.16	1.17	-	-	0.02

## Supplementary Materials

The following are available online, Table S1: Ion transitions used for quantification (MRM1) and confirmation (MRM2), and dwell time, cone voltage, and collision energy for mass spectrometry settings for different pesticide compounds, Table S2: Chemical structure, and acute oral and contact LD50 values of pesticide compounds in honeybees, Table S3: Limits of determination and quantification (LOD and LOQ), and linear ranges, linear regression equations, and linearities of the method for different pesticide compounds.
