# Supplementary material for: Evaluation of Highly Detectable Pesticides Sprayed in Brassica napus L.: Degradation Behavior and Risk Assessment for Honeybees
Source: Molecules. 2018 Sep 27;23(10):2482. doi: 10.3390/molecules23102482 (PMC6222740; doi:10.3390/molecules23102482)
Supplement: Supplementary file 1 [file molecules-23-02482-s001.pdf]

Table S1. Ion transitions used for quantification (MRM1) and confirmation (MRM2), and dwell time, cone voltage, and collision energy for mass spectrometry settings for different pesticide compounds.

Compound	Transitions	Dwell time (ms)	Cone voltage (V)	Collision energy (eV)
Carbendazim	Quantification ion 192.1 >160.1	28	24	18
	Confirmation ion 192.1 >132.1		24	28
Thiamethoxam	Quantification ion 292.1 >210.9	44	18	12
	Confirmation ion 292.1 >181		18	24
Imidacloprid	Quantification ion 256.1 >209.1	28	23	15
	Confirmation ion 256.1 >175.1		23	20
Acetamiprid	Quantification ion 223 >126	28	23	20
	Confirmation ion 223 >56.1		23	15
Pyrimethanil	Quantification ion 200.2 >107	22	42	24
	Confirmation ion 200.2 >82		42	24
Procholraz	Quantification ion 376 >308	8	20	15
	Confirmation ion 376 >266		20	15
Chlorpyrifos	Quantification ion 350 >97	22	27	32
	Confirmation ion 350 >198		27	20
Fenpropathrin	Quantification ion 350.1 >97	22	15	34
	Confirmation ion 350.1 >125		15	14

Table S2. Chemical structure, and acute oral and contact LD50 values of pesticide

compounds in honeybees.[a]

Compound	Chemical structure	Acute oral LD50 (ng a.i./ bee)	Acute contact LD50 (ng a.i./ bee)
imidacloprid	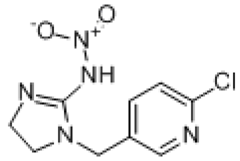	1.3	6.1
thiamethoxam	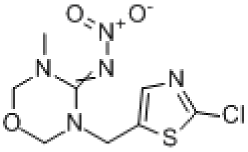	5.0	25
acetamiprid	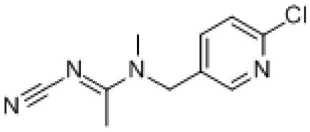	1.4×10^4	7.9×10^3
fenpropathrin	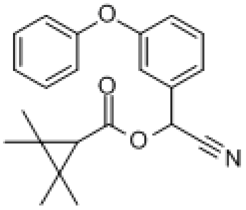	50	6.4×10^2
chlorpyrifos	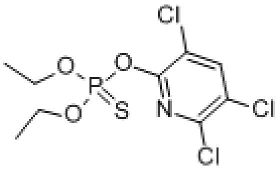	2.4×10^2	72
carbendazim	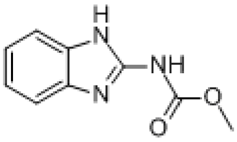	$>5 \times 10^4$	-
prochloraz	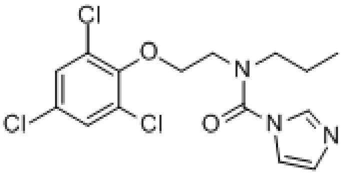	$>1 \times 10^2$	$>1 \times 10^2$
pyrimethanil	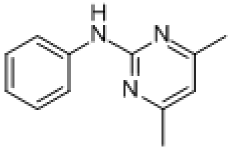	5×10^4	6×10^4

[a] Sources: Pesticide Manual (2009); ECOTOX and AGRITOX databases.

Table S3. Limits of determination and quantification (LOD and LOQ), and linear ranges, linear regression equations, and linearities of the method for different pesticide compounds.

Compound	LOD (ng/g)	LOQ (ng/g)	Linear range (ng/g)	Linear regression equation	Linearity
Carbendazim	0.1064	0.3191		Y = 941.6X + 11.03	0.9993
Thiamethoxam	0.0028	0.0084		Y = 66.00X + 134.2	0.9975
Imidacloprid	0.0809	0.2427		Y = 71.91X − 48.57	0.9996
Acetamiprid	0.0114	0.0343	1–100	Y = 645.1X + 279.1	0.9986
Pyrimethanil	0.0145	0.0435		Y = 1810X − 63.22	0.9999
Procholraz	0.0166	0.0499		Y = 587.4X − 606.3	0.9902
Chlorpyrifos	0.0638	0.1914		Y = 327.6X + 67.46	0.9995
Fenpropathrin	0.0433	0.1300		Y = 407.6X + 67.03	0.9999
